# Father’s involvement associated with rural children’s depression and anxiety: A large-scale analysis based on data from seven provinces in China

**DOI:** 10.1017/gmh.2024.70

**Published:** 2024-08-27

**Authors:** Jian Jiang, Xuwei Tang, Zhifeng Lin, Yulan Lin, Zhijian Hu

**Affiliations:** School of Public Health, Fujian Medical University, Fuzhou 350122, Fujian Province, China

**Keywords:** father involvement, depression, anxiety, children and adolescents, a large-scale analysis

## Abstract

To investigate the relationship between father involvement in parenting and mental health problems among children and adolescents in rural China. The Rural Children’s Mental Health dataset includes mental health information from 2,489 children and adolescents aged 5–16 in seven provinces in China. The relationship between father involvement in children and adolescents depression risk and anxiety was analyzed by Spearman’s correlation analysis, logistic regression analysis, and restricted cubic spline. Father involvement was significantly and negatively associated with depression scores (*r* = −0.38, *P* < 0.001) and anxiety scores (*r* = −0.18, *P* < 0.001) in rural Chinese children and adolescents. Both multivariate models indicate that the highest level of father involvement has a protective effect on the risk of depression among children and adolescents (*OR* = 0.268 and 0.303, 95% *CI*: 0.149~0.483 and 0.144~0.636), while the association with anxiety risk is only significant in the multivariate model 1 (*OR* = 0.570, 95% *CI*: 0.363~0.896). Father involvement is a protective factor for the risk of depression among children and adolescents in rural China. The level of father involvement should be increased, and active participation should be encouraged to reduce the risk of depression in their children and to further promote the mental health of children and adolescents in China.

## Graphical abstract

**Figure:**
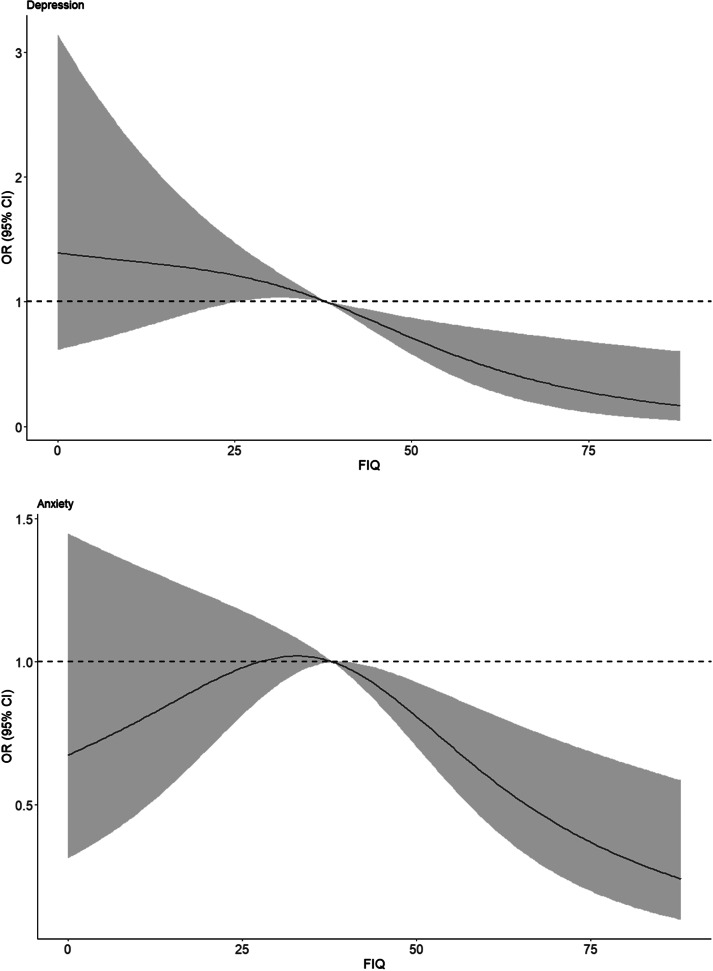

## Impact statement

Mental health problems in children and adolescents have become one of the most important public health challenges of the 21st century, with negative mood disorders such as depression and anxiety being the most common and prevalent mental health problems, with family environments such as father involvement being an important influencing factor. However, there is still relatively little research on the correlation between father involvement and mental health problems among children and adolescents in rural China. The current study, using information from a mental health database of rural children in China, analyses the relationship between father involvement and depressive and anxiety symptoms among children and adolescents in rural China. This study demonstrates that father involvement is a protective factor for the risk of depression among children and adolescents in rural China. The level of father involvement should be increased, and active participation should be encouraged to reduce the risk of depression in their children and to further promote the mental health of children and adolescents in China.

## Introduction

Mental health, especially mood disorders such as depression and anxiety in children and adolescents is acknowledged to be of considerable concern. The global prevalence of depression and anxiety among children and adolescents is estimated to be 13.4% (Bradshaw et al., 2021) With rapid economic and social development, the prevalence of mental health problems, such as depression and anxiety, among children and adolescents in China has also been on the rise. Some studies have reported that the prevalence of depression and anxiety among Chinese children and adolescents is 24.6% and 35.7%, respectively (Dong et al., 2023) while the prevalence among rural left-behind children is 51.5% and 57.6% (Cui et al., 2021) These emotional problems are highly correlated with a number of negative outcomes, including learning difficulties, substance abuse, bullying, stigmatization, employment difficulties in adulthood, low incomes, self-harm and suicide (Donato et al., 2021; Schlack et al., 2021; Viswanathan et al., 2022a, 2022b). Thus, understanding and investigating the factors that influence depression and anxiety in children and adolescents is crucial and can provide excellent opportunities for early intervention and treatment.

Parenting theory (Del Barrio et al., 2016) and attachment theoretical frameworks (Peng et al., 2021) argue that the family environment is the primary place where people live and that family structure (Fritzell et al., 2019), parental mental health status (Raskin et al., 2015), family migration (Lu et al., 2020), parent–child relationships (Brouillard et al., 2018), parental education (Wille et al., 2008) and parenting styles (Fagan, 2022) are key influences on depression and anxiety in children and adolescents. Although there is a growing clarity of knowledge about how family contexts contribute to children and adolescents mental health, most of these studies have focused on the role of mothers, often ignoring the function of fathers’ roles in family parenting involvement (Panter-Brick et al., 2014), and even studies on fathers have considered them as mediators of their children’s mental health (Ibrahim et al., 2017) or as emotion regulators (Shenaar-Golan et al., 2021) and have failed to recognize the unique and important roles of the father’s role in parenting involvement.

Currently, the role of fathers has changed to become important participants in caring for family and raising children (Nettelbladt et al., 1980). Father involvement has been defined as the emotional, cognitive and behavioral guidance and investment that fathers give to their children in order for them to be able to grow up in a healthy way (Coleman and Garfield, 2004), and it is a complex structure that includes three dimensions of accessibility, interaction and responsibility (Sarkadi et al., 2008), not only providing financial support but also the interaction, caring, support and attitudes toward their children, their reactions to their children’s emotions, and their sense of security in their own roles (Opondo et al., 2017). In light of all this, understanding the role of father’s involvement in children and adolescents mental health during adolescence is critical (Dadds et al., 2018; Garcia et al., 2022). It has been noted that early father involvement can moderate children’s susceptibility to mental health problems in adolescence and reduce the severity of mental health problems (Boyce et al., 2006), while father absence may lead to depression (Markowitz and Ryan, 2016), anxiety (Shenaar-Golan et al., 2021) and delinquency in children and adolescents (Culpin et al., 2013). The active involvement of fathers in parenting behaviors can significantly improve children’s emotional cognitive and social development, such as depression and anxiety, and achieve better physical and mental health (Allport et al., 2018). The father-child relationship may also influence the motivation and efforts of children and adolescents to seek mental health services (Reeb and Conger, 2011).

The relationship between father involvement in parenting behaviors and their children’s mental health has also attracted widespread attention in China. Studies in Chinese populations have shown that children with active fathers have better mental health (Jiang et al., 2023), and parental migration puts both migrant and left-behind children at greater risk for mental health problems (Lu et al., 2020). In addition, fathers’ over-involvement in parenting increases children’s anxiety (Leung, 2021). Unfortunately, despite the numerous studies conducted so far, most research exploring the relationship between father involvement and the mental health of children and adolescents within the context of Chinese culture still exhibits several shortcomings. First, although there have been a few studies on the impact of fathers’ participation on children’s mental health, most of the analyses have included paternal parenting as a covariate. Few studies have considered fathers’ participation as an independent factor in the family parenting environment and specifically focused on the relationship between paternal parenting and children’s mental health issues. Second, while the positive and negative effects of father involvement in children’s mental health have been examined, the extent and level of father involvement have not been further analyzed. Third, previous studies have mostly combined children and adolescents in their analysis and did not separate these two differently represented groups, especially compared to urban areas, rural fathers tend to have lower educational levels, more unstable jobs, less parenting knowledge and skills and spend less time with their families (Zhao et al., 2014; Huang et al., 2015). Therefore, there is a greater need to study the relationship between fathers’ involvement and their children’s mental health.

To address this research gap, this study conducted an analysis of mental health data from rural children and adolescents in seven provinces of China, emphasizing the relationship between fathers’ parenting behaviors and the mental health of children and adolescents in rural China. Based on the scores from a questionnaire on fathers’ involvement, the study stratified their participation in parenting and explored its impact as an independent variable on their children’s depression and anxiety. This analysis aims to provide policy references for improving and enhancing the mental health of rural children and adolescents.

## Methods

### Data sources

This was an observational study based on a public mental health database of Chinese rural children (Fang et al., 2022). Of note, this mental health database is a subset of the Chinese National Mental Health Database (https://cmhr.psych.cn/m/), which is an open, freely accessible dataset. This dataset is a questionnaire survey of mental health problems and related influencing factors among children and adolescents aged 5–16 years old in rural China using a convenience sampling methodology, and the inclusion criteria for the survey are students in grades 1–6 of elementary school in rural China. In general, researchers carried out a questionnaire survey between March 2021 and May 2021 for 16 rural elementary schools in seven provinces in China, including Anhui, Gansu, Guangdong, Heilongjiang, Hubei, Hunan and Sichuan provinces.

**Figure 1.**
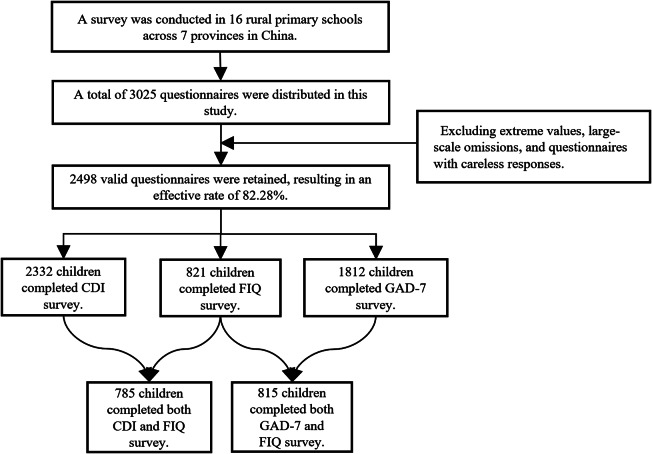
Flowchart of recruitment of study participants. *Note:* A total of 2,332 completed the Child Depression Inventory (CDI), 1,812 completed the Generalized Anxiety Disorder 7-item (GAD-7), 821 completed the Father Involvement Questionnaire (FIQ), 785 completed the Child Depression Inventory (CDI) and Fathe Involvement Questionnaire (FIQ), 815 completed the Generalized Anxiety Disorder 7-item (GAD-7) and Father Involvement Questionnaire (FIQ).

A total of 2,498 out of 3,025 distributed questionnaires were included in the final analysis after excluding extreme values, large-scale omissions and questionnaire with invalid responses. Figure 1 shows the flow chart for the selection of study population. A total of 785 students completed both CDI and FIQ survey, while 815 students completed both GAD-7 and FIQ surveys. These two population are the main study populations for the current study.

### Measurement tools

#### Father involvement

The survey used the Father Involvement Questionnaire (FIQ) to measure the level of fathers’ parenting involvement (Wu et al., 2015), which has 22 questions on a 5-point Likert scale from 0 (never) to 4 (always), with scores for all questions summed up and scores ranging from 0 to 88, and higher scores indicating higher levels of involvement in parenting by fathers. The FIQ is organized around the three dimensions of accessibility, interaction and responsibility, there are nine items for accessibility, seven items for interaction, and six items for responsibility (Supplementary Appendix). The reliability and validity of the FIQ have been tested (Wu et al., 2015), and the internal consistency coefficient in this survey was 0.94. For the purpose of research and analysis, FIQ scores were categorized into four groups according to quartiles: low, middle-low, middle-high and high.

#### Depression levels

Depressive symptoms in rural children and adolescents in the last two weeks were assessed using the Child Depression Inventory (CDI) (Kovacs, 1985), a 27-question scale using a 3-point Likert scale from 0 to 2, where each option consists of statements describing varying degrees of depressive symptoms: 0 points for “general reaction,” 1 point for “major depressive symptoms,” and 2 points for “severe depressive symptoms,” and scores for all the questions were summed up, which range from 0 to 54 points, with higher scores indicating higher levels of depression. A score of 0–19 is considered low risk for depression and a score higher than 19 is considered high risk for depression. The internal consistency coefficient for this scale in this survey was 0.84.

#### Anxiety symptoms

Anxiety symptoms in children and adolescents were measured over a 2-week period using the Generalized Anxiety Disorder 7-item (GAD-7) (Spitzer et al., 2006). The GAD-7 is set up with seven questions, each scored on a 4-point Likert scale from 0 (not at all) to 3 (almost every day), and all question scores are summed for a range of scores from 0 to 21, which are categorized into four dimensions based on the scores: no anxiety (0–4 points), mild anxiety (5–9 points), moderate anxiety (10–14 points) and severe anxiety (15–21 points). Higher scores indicate higher levels of anxiety in individuals. The internal consistency coefficient of the scale was 0.81 in this survey.

#### Background information

The survey also synchronized the collection of basic background information such as gender, age, family situation and parents’ education level of the participants.

### Statistical analysis

First, background characteristics of the children and adolescents were described, using frequencies and percentages to describe categorical variables. Depression and anxiety scores were found to be nonnormally distributed by normality tests, and the median and interquartile range (IQR) were used to describe the depression and anxiety risk scores of the different subgroups.

Second, Spearman correlation analyses were conducted to test the relationship between children and adolescents depression scores and anxiety scores and father involvement. To improve the robustness of the results, we used logistic regression to validate the effect of the level of father involvement on mental health problems by incorporating relevant covariates based on a univariate model and two separate multivariate models constructed for depression and anxiety risk.

Finally, restricted cubic spline plots with three knots were drawn, using the median FIQ score of 38 as a reference, to analyze the dose-response relationship between fathers’ parenting investment and the risk of depression and anxiety risk among children and adolescents. Restricted cubic spline plots were constructed for the relationship between fathers’ parenting investment and depression risk scores as well as anxiety risk scores, respectively.

SPSS 27.0 (SPSS Inc., Chicago IL) was used for descriptive statistics, Spearman correlation analysis, and logistic regression analysis. The rms package of R4.2.3 software was used for restricted cubic spline analysis. All statistical analyses were performed using two-sided tests with a test level of *α* = 0.05.

### Ethics

The data used in the study are open access data, which have been cleaned and do not involve any personally identifiable information or privacy, so there are no ethical issues with the study.

## Results

Of 2,498 valid questionnaires, 1,277 (51.1%) were female and 49 (2%) did not report gender; age ranged from 5 to 16 years (M = 10.67, SD = 1.71) and 301 (12%) did not report age; 51.2% and 58.8% reported living with a biological father or mother, respectively; 67.2% reported a harmonious parental relationship; more than one-third had a parent with a middle school education level or lower; 11.1% reported having no siblings; the median and IQR of depression risk scores was 13 (9–19), the median and IQR of anxiety risk scores was 6 (3–10), and the median and IQR of father involvement scores was interquartile spacing of 38 (23–52). Detailed background information about the participants is presented in Table 1.

**Table 1.** Background information and depression, anxiety and FIQ scores of the study population

Variable	*N*	%	*n*_1_	%	*n*_2_	%
Gender						
Male	1,172	46.9	357	45.5	369	45.3
Female	1,277	51.1	414	52.7	433	53.1
Not reported	49	2.0	14	1.8	13	1.6
Age						
5–9	534	21.4	2	0.3	2	0.2
10–11	877	35.1	149	19.0	151	18.5
12–16	786	31.5	615	78.3	642	78.8
Not reported	301	12.0	19	2.4	20	2.5
Accompanied by biological father						
Yes	1,278	51.2	449	57.2	466	57.2
No	1,147	45.9	327	41.7	336	41.2
Not reported	73	2.9	9	1.1	13	1.6
Accompanied by biological mother						
Yes	1,470	58.8	517	65.9	538	66.0
No	953	38.2	259	33.0	264	32.4
Not reported	75	3.0	9	1.1	13	1.6
Parental relations						
Harmony	1,567	62.7	559	71.2	579	71.0
General	539	21.6	133	16.9.	136	16.7
Disharmony	303	12.1	79	10.1	83	10.2
Not reported	89	3.6	14	1.8	17	2.1
Father’s education level						
Lower secondary and below	897	36.0	409	51.6	418	51.3
High school/vocational high school/secondary school/college	444	17.7	156	19.9	162	19.9
University and above	181	7.2	30	3.8	32	3.9
Not reported	976	39.1	194	24.7	203	24.9
Mother’s education level						
Lower secondary and below	885	35.4	405	51.6	422	51.8
High school/vocational high school/secondary school/college	432	17.3	156	19.9	157	19.3
University and above	218	8.7	32	4.1	34	4.2
Not reported	96.3	38.6	192	24.5	202	24.8
One–child						
Yes	278	11.1	103	13.1	106	13.0
No	2,117	84.7	670	85.4	693	85.0
No reported	103	4.1	12	1.5	16	2.0
Depression risk						
Yes	571	22.9	199	25.4	198	24.3
No	1,761	70.5	586	74.6	583	71.5
Not reported	166	6.6	0	0	34	4.2
Depression score (median [IQR])	13[9–19]		13[9–20]		13[9–20]	
Anxiety level						
No	729	29.2	346	44.1	364	44.7
Mild	617	24.7	243	31.0	250	30.7
Moderate	376	15.1	144	18.3	153	18.8
Severe	90	3.6	48	6.1	48	5.9
Not reported	686	27.5	4	0.5	0	0
Anxiety score (median [IQR])	6[3–10]		5[2–9]		5[2–9]	
FIQ score (median [IQR])	38[23–52]		38[23–53]		38[23–53]	

*Note:* Data are from “A mental health database of rural children.” Categorical variables are given as *n* and %, and continuous variables are given as the median ([IQR]), where *N* is information on 2,498 individuals, *n*_1_ indicates information on 785 individuals who completed the CDI and FIQ, and *n*_2_ indicates information on 815 individuals who completed the GAD-7 and FIQ. CDI, Child Depression Inventory; GAD-7, Generalized Anxiety Disorder 7-item; FIQ, Father Involvement Questionnaire; IQR, Interquartile Range.

**Figure 2.**
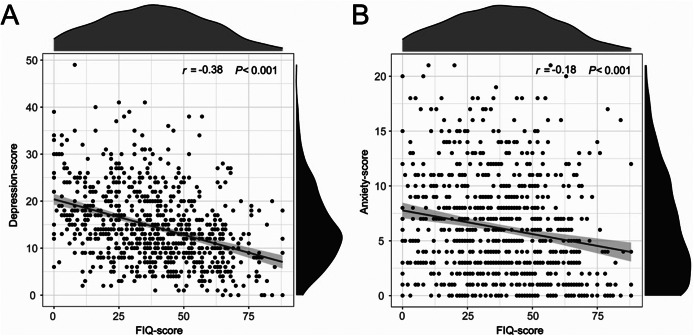
Spearman’s correlation analysis for father involvement and depression and anxiety scores among Chinese rural children and adolescents. *Note:* (A) The relationship between depression scores and father involvement and (B) the relationship between anxiety scores and father involvement. FIQ, Father Involvement Questionnaire.

The depression and anxiety risk scores were skewed by the normality test. The association of background information with depression risk and anxiety risk was analyzed using the rank sum test, which showed that rural children and adolescents depression risk and anxiety risk were significantly different on factors related to age, parental relationship, and biological parent’s companionship (Supplementary Table). A Spearman correlation analysis was conducted to examine the relationship between paternal parenting investment and the scores of depression and anxiety among rural children and adolescents, testing the association between paternal parenting investment and the risk of depression and anxiety risk. Figure 2 illustrates the results of Spearman’s correlation analysis, with father involvement having a significant negative association with both depression scores (*r* = −0.38, *P* < 0.001) and anxiety scores (*r* = −0.18, *P* < 0.001).

Subsequently, logistic regression analyses were performed on 785 individuals who completed both the CDI and the FIQ to validate the relationship between depression risk and father involvement in rural children and adolescents. The variance inflation factor for each variable is less than 7.5 (VIF = 1.013 ~ 1.626), suggesting that there is no large covariance between the variables. The results of the logistic regression analysis showed that the aspects of parental relationship, biological father’s companionship, biological mother’s companionship and father’s level of involvement were significantly related to the depression risk score in the univariate analysis.

To investigate the impact of father involvement, a multifactorial model 1 was constructed by incorporating statistically significant factors from the univariate analysis as independent variables in the multifactorial analysis. After adjusting for moderators, the results showed that among rural children and adolescents, higher levels of father involvement predicted a lower risk of depression compared to low levels. Using the lowest quartile group of the FIQ quartiles as the reference, the *OR* (95% *CI*) for the other three groups, from low to high, were 0.929 (0.595 ~ 1.451), 0.599 (0.369 ~ 0.972), and 0.268 (0.149 ~ 0.483), respectively, *P* for trend ≤ 0.001. Subsequently, a multivariate model 2 was constructed utilizing all factors as independent variables, showing that fathers with higher levels of involvement faced a lower risk of depression than fathers with lower levels of involvement in parenting. Using the lowest quartile group after grouping the FIQ quartiles as a reference, the *OR* (95% *CI*) of the other three groups, from low to high, were 0.799 (0.442 ~ 1.444), 0.787 (0.428 ~ 1.447) and 0.303 (0.144 ~ 0.636), *P* for trend = 0.013 (Table 2). This suggests that more positive father involvement is a protective factor in children and adolescents depression problems.

**Table 2.** Logistic regression model of the relationship between depression risk scores and background information such as FIQ score

Variable	Univariate analysis		Multivariable analysis model 1		Multivariable analysis model 2		
	*OR* (95% *CI*)	*P*	*OR* (95% *CI*)	*P*	*OR* (95% *CI*)	*P*	VIF
Gender		0.928				0.022	1.039
Male	1.000 (reference)				1.000 (reference)		
Female	0.991 (0.819, 1.200)				1.696 (1.079, 2.665)		
Age		0.377				0.274	1.013
5–10	1.000 (reference)				1.000 (reference)		
11–16	1.097 (0.893, 1.347)				0.357 (0.056, 2.263)		
Parental relations		<0.001		<0.001		<0.001	1.156
Harmony	1.000 (reference)		1.000 (reference)		1.000 (reference)		
General	2.345 (1.867, 2.945)		2.401 (1.564, 3.687)		3.764 (2.169, 6.531)		
Disharmony	4.101 (3.113, 5.403)		2.917 (1.723, 4.937)		3.649 (1.853, 7.187)	0.341	1.626
Accompanied by biological father		<0.001		0.091			
No	1.000 (reference)		1.000 (reference)		1.000 (reference)		
Yes	0.706 (0.583, 0.856)		0.698 (0.459, 1.060)		0.767 (0.444, 1.324)	0.950	1.615
Accompanied by biological mother		0.004		0.889			
No	1.000 (reference)		1.000 (reference)		1.000 (reference)		
Yes	0.754 (0.621, 0.916)		1.031 (0.670, 1.587)		1.019 (0.571, 1.818)	0.336	1.151
One–child		0.058				0.162	
No	1.000 (reference)				1.000 (reference)		
Yes	0.730 (0.527, 1.010)				1.606 (0.826, 3.119)		
Father’s education level		0.871				0.383	1.545
Lower secondary and below	1.000 (reference)				1.000 (reference)		
High school/vocational high school/secondary school/college	0.928 (0.702, 1.227)				1.468 (0.821, 2.626)		
University and above	0.983 (0.667, 1.449)				1.673 (0.501, 5.584)		
Mother’s education level		0.282				0.174	1.555
Lower secondary and below	1.000 (reference)				1.000 (reference)		
High school/vocational high school/secondary school/college	0.793 (0.596, 1.055)				0.583 (0.319, 1.066)		
University and above	0.930 (0.650, 1.333)				0.495 (0.139, 1.757)		
FIQ score		<0.001		<0.001		0.013	1.199
Low	1.000 (reference)		1.000 (reference)		1.000 (reference)		
Medium–low	0.723 (0.478, 1.092)		0.929 (0.595, 1.451)		0.799 (0.442, 1.444)		
Medium–high	0.425 (0.270, 0.667)		0.599 (0.369, 0.972)		0.787 (0.428, 1.447)		
High	0.166 (0.095, 0.288)		0.268 (0.149, 0.483)		0.303 (0.144, 0.636)		

*Note:* Data are from “A mental health database of rural children.” A logistic regression analysis of the relationship between the background characteristics of the children and adolescents and the risk of depression was conducted, and a multivariate model was constructed based on univariate analysis. The variables included in model 1 were parental relationship, whether the biological father was accompanied, whether the biological mother was accompanied and the level of father involvement. The variables included in Model 2 were gender, age, parental relationship, whether the biological father was accompanied, whether the biological mother was accompanied, whether the stepfather was accompanied, whether the stepmother was accompanied, whether the child was an only child, the father’s education level, the mother’s education level and the level of father involvement. *P* ≤ 0.05 was considered significant. FIQ, Father Involvement Questionnaire.

Additionally, logistic regression analyses were conducted on 815 individuals who completed both the GAD-7 and the FIQ to validate the relationship between anxiety risk and father involvement in rural children and adolescents. The variance inflation factor for each variable is less than 7.5 (VIF = 1.012 ~ 1.628), suggesting that there is no large covariance between the variables. Consistent with the analysis of depression risk, univariate and multivariate analyses were performed to establish the respective multivariate models 1 and multivariate models 2. Regarding anxiety risk scores, only the highest level of father involvement in the univariate model (*OR* = 0.387, 95% *CI*: 0.259~0.577) and multivariate model 1 (*OR* = 0.570, 95% *CI*: 0.363~0.896) was associated with lower anxiety risk among children and adolescents. The association between other levels of father involvement and anxiety risk in children and adolescents was not statistically significant (Table 3).

**Table 3.** Logistic regression model of the relationship between anxiety risk scores and background information such as FIQ score

Variable	Univariate analysis		Multivariable analysis model 1		Multivariable analysis model 2		VIF
	*OR* (95% *CI*)	*P*	*OR* (95% *CI*)	*P*	*OR* (95% *CI*)	*P*	
Gender		0.033		0.102		0.034	1.036
Male	1.000 (reference)		1.000 (reference)		1.000 (reference)		
Female	1.229 (1.017, 1.486)		1.283 (0.952, 1.728)		1.493 (1.03, 2.165)		
Age		0.010		0.085		0.220	1.012
5–10	1.000 (reference)		1.000 (reference)		1.000 (reference)		
11–16	0.753 (0.607, 0.935)		0.157 (0.019, 1.292)		0.245 (0.026, 2.327)		
Parental relations		<0.001		<0.001		<0.001	1.164
Harmony	1.000 (reference)		1.000 (reference)		1.000 (reference)		
General	2.308 (1.785, 2.984)		2.564 (1.643, 4.002)		4.277 (2.346, 7.796)		
Disharmony	2.265 (1.586, 3.236)		2.044 (1.192, 3.507)		3.22 (1.588, 6.528)		
Accompanied by biological father		0.005		0.049		0.224	1.628
No	1.000 (reference)		1.000 (reference)		1.000 (reference)		
Yes	0.760 (0.627, 0.921)		0.690 (0.477, 0.999)		0.744 (0.462, 1.199)		
Accompanied by biological mother		0.007		0.353		0.888	1.619
No	1.000 (reference)		1.000 (reference)		1.000 (reference)		
Yes	0.757 (0.619, 0.925)		1.201 (0.816, 1.768)		1.038 (0.622, 1.731)		
One–child		0.163				0.625	1.034
No	1.000 (reference)				1.000 (reference)		
Yes	0.808 (0.600, 1.090)				0.869 (0.494, 1.528)		
Father’s education level		0.978				0.764	1.551
Lower secondary and below	1.000 (reference)				1.000 (reference)		
High school/vocational high school/secondary school/college	1.012 (0.783, 1.307)				0.852 (0.527, 1.377)		
University and above	0.967 (0.654, 1.43)				0.776 (0.289, 2.084)		
Mother’s education level		0.899				0.865	1.560
Lower secondary and below	1.000 (reference)				1.000 (reference)		
High school/vocational high school/secondary school/college	1.065 (0.816, 1.389)				1.019 (0.628, 1.655)		
University and above	1.020 (0.709, 1.468)				0.775 (0.281, 2.137)		
FIQ score		<0.001		0.036		0.080	1.201
Low	1.000 reference)		1.000 (reference)		1.000 (reference)		
Medium–low	0.777 (0.522, 1.158)		0.962 (0.623, 1.484)		1.096 (0.622, 1.930)		
Medium–high	0.683 (0.457, 1.019)		0.938 (0.604, 1.458)		1.338 (0.763, 2.346)		
High	0.387 (0.259, 0.577)		0.570 (0.363, 0.896)		0.703 (0.396, 1.247)		

*Note:* Data are from “A mental health database of rural children.” A logistic regression analysis of the relationship between the background characteristics of the children and adolescents and their levels of anxiety was conducted, and a multivariate model was constructed based on a univariate analysis. The variables included in Model 1 for analysis were gender, age, parental relationship, whether the biological father was accompanied, whether the biological mother was accompanied and the level of father involvement. The variables included in Model 2 were sex, age, parental relationship, whether the child was accompanied by a biological father, whether the child was accompanied by a biological mother, whether the child was accompanied by a stepfather, whether the child was accompanied by a stepmother, whether the child was an only child, the father’s education level, the mother’s education level and the level of father involvement. *P* ≤ 0.05 was considered significant. FIQ= Father Involvement Questionnaire.

**Figure 3.**
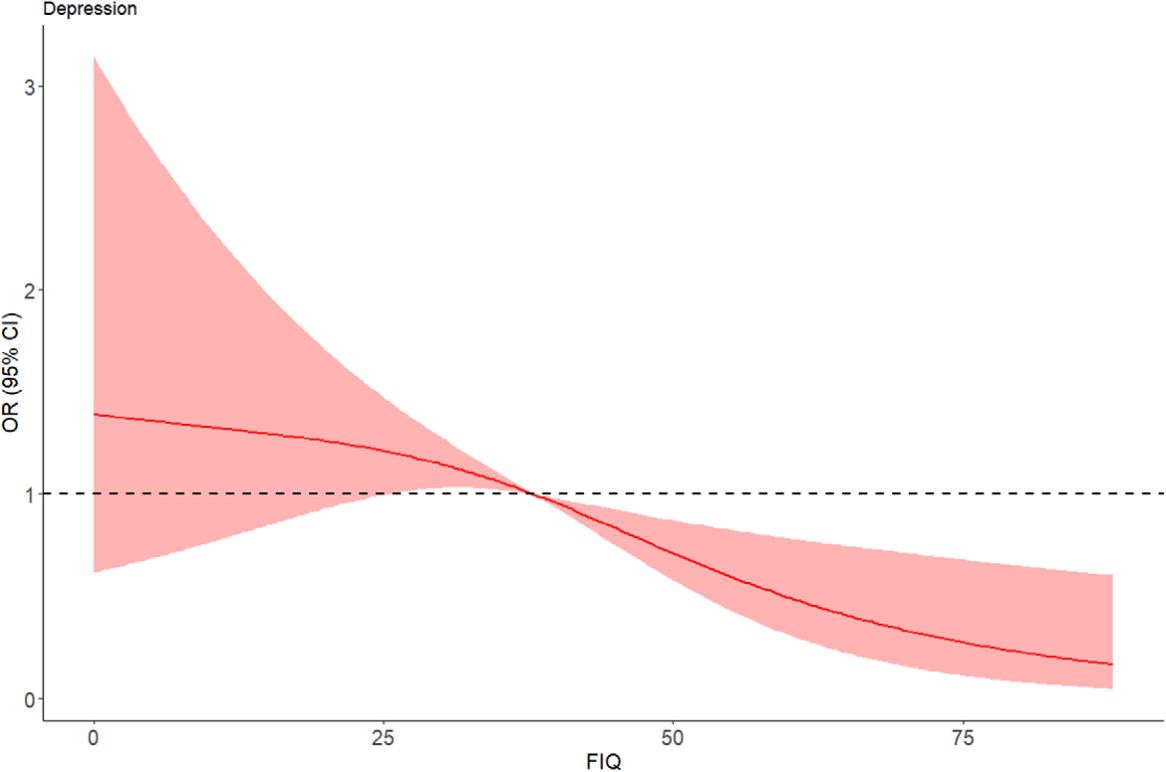
Quantitative-effective relationship between FIQ score and risk of depression. *Note:* The red line in the graph indicates the trend of the fit between the FIQ score and the risk of depression, and the red area indicates the 95% CI of the fitted curve. FIQ, Father Involvement Questionnaire.

**Figure 4.**
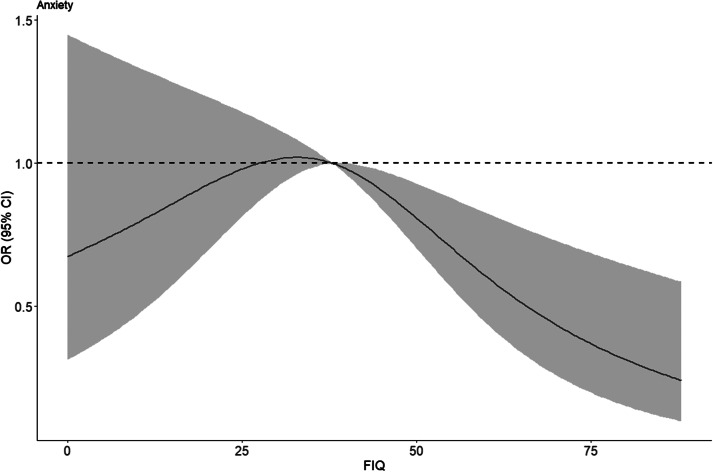
Quantitative-effective relationship between FIQ score and risk of anxiety. *Note:* The red line in the graph indicates the trend of the fit between the FIQ score and risk of anxiety, and the red area indicates the 95% CI of the fitted curve. FIQ, Father Involvement Questionnaire.

Finally, restricted cubic splines were plotted at three knots, demonstrating the quantitative validity of the relationship between father involvement and the depression risk score (Figure 3) as well as the anxiety risk score (Figure 4) among rural children and adolescents, respectively. Overall, the risk of depression and anxiety risk both tended to decrease gradually as father involvement increased. Although medium-low levels of father involvement did not show a statistically significant relationship with depression risk and anxiety risk, the red trend and red areas in the graph indicate that medium-high levels of father involvement significantly reduce children and adolescents depression risk, particularly for anxiety. Father involvement also exerted a significant protective effect on the anxiety risk of children and adolescents once the score exceeded 38.

## Discussion

In the past four decades, with drastic social transformation, rapid economic development, and the impact of East–West cultural exchanges, Chinese children and adolescents are at a higher risk of mental health problems, especially those in rural areas of China, who face lower socioeconomic status, poorer education, more backward growth environments, fewer parental companions, and higher symptoms of depression and anxiety, which urgently need more attention and research (Fellmeth et al., 2018; Tang et al., 2018). To the best of our knowledge, this is one of the few to discuss father involvement and the mental health of children and adolescents in rural China. A nationally representative survey was utilized to conduct secondary analyses of data collected from children and adolescents aged 5–16 years attending 16 rural elementary schools across seven provinces in China. The aim of these analyses was to reveal the association between Chinese father involvement and their children’s risk of depression and levels of anxiety. The study found that father involvement has a protective effect on the mental health problems such as depression and anxiety among rural children and adolescents.

Considering conditions such as economic and social status, family composition, parenting styles, and cultural background, previous studies have emphasized the role of mothers (Briscoe et al., 2019; Wu et al., 2022), and the influence of fathers on their children’s mental health tends to be a factor that is easily overlooked (Jeong et al., 2021). Although evidence suggests that positive father involvement can have a unique and lasting impact on the development of children and adolescents mental health and the treatment of mental health problems, research on involvement still lags behind that of mothers, and more attention needs to be paid to the relationship between father involvement and children and adolescents mental health problems in the current setting, especially when designing and implementing intervention programs (Gonzalez et al., 2023).

The current study evaluates the level of father involvement investment from three dimensions: interactivity, accessibility and responsibility (Wu et al., 2015) and divided father involvement into four levels: low, medium-low, medium-high and high based on the scores. The results showed that the level of father involvement was significant in both the risk of depression and anxiety scores of the children and adolescents. The higher the level of father involvement is, the lower the risk of depression and anxiety in the children. This is similar to the findings of other researchers (Yap et al., 2014; Suh et al., 2016), whose studies also suggest that among the family environment factors, greater father input and involvement and better father-child parenting are protective factors for children and adolescents mental health.

Attachment theory suggests that fathers play an irreplaceable role in children’s development and that the father-child relationship is relevant to the mental health and emotional development of children and adolescents (Peng et al., 2021). The analysis revealed that the higher the father involvement, the better the children and adolescents psychological adjustment and the lower the risk of depression, along with the increasing level of father involvement, the children’s risk of depression is decreasing, and father involvement is a protective factor for children and adolescents depression. This is consistent with previous findings that more positive father involvement is effective in preventing psychological distress in children and adolescents (Flouri, 2008) and can enhance resilience and frustration tolerance (Liu et al., 2023), thereby reducing the risk of depression and promoting good mental health status.

The association between anxiety risk and the degree of father involvement is somewhat inconsistent in the data analysis. Only the highest level of father involvement in the univariate model and multivariate model 1 showed a protective effect on the anxiety risk of children and adolescents. The dimensions of parenting behavior and the level of involvement have both positive and negative effects on the mental health of children and adolescents (Weitkamp and Seiffge-Krenke, 2019). Despite the evidence from previous studies that the active involvement of fathers significantly lowers children and adolescents anxiety symptoms (Yap et al., 2014; Ibrahim et al., 2017), it has also been suggested that excessive parenting behaviors by fathers increase children’s anxiety risk (Jiang et al., 2023), which seems to be confirmed by our results, and that fathers’ excessive involvement in parenting behavior may be perceived by children in adolescence as a restriction on the individual’s pursuit of freedom and independence, leading to tensions and conflicts in the parent–child relationship, which may affect psychological well-being (Peng et al., 2021). However, explaining such behaviors should be done with some caution, especially in rural Chinese families, where the interaction mechanism between excessive father-child involvement in parenting behaviors and children’s tensions and conflicts is unclear.

What is clear, however, is that father involvement is a modifiable factor in the family environment (Coleman et al., 2004; Tang et al., 2018; Gonzalez et al., 2023). The present study demonstrates that father involvement is an important influence on children’s mental health; it is thus important to emphasize the role of fathers in the family environment, in addition to the maintenance of a family atmosphere and the creation of good family relationships, in the mental health of children and adolescents in rural areas. The findings provide a new perspective on interventions for children and adolescents mental health, High levels of father involvement predict lower risk of depression and anxiety in their children, suggesting that interventions to promote children and adolescents mental health can be made from the fathers’ perspective, which provides a basis for more targeted public health policies, prevention strategies and interventions to promote children and adolescents mental health issues.

### Limitations and future research

This study has several limitations. First, the data used in this study were collected through non-random sampling methods, consequently, there may be sampling bias. Second, these findings were derived from self-reported data. Although this is a common methodology used in research on children and adolescents mental health issues, there is still the possibility of individual self-perceived bias; for instance, individuals with poorer mental health may report lower levels of father involvement, leading to differences in results. Future research should combine multiple reporting sources, including parent-reported scales, teacher-reported scales, and structured interviews for a comprehensive analysis. Thirdly, the study did not include relevant variables related to the mother, thus there was no way to analyze the relationship between other family variables characteristics, such as motherhood, and the FQI. Fourth, although FQI is a continuous scoring questionnaire divided into three dimensions, current evidence does not support analyzing the three dimensions as independent variables. Future studies should aim to obtain more evidence for each dimension. Finally, as this study is based on cross-sectional survey data, it is not possible to establish a control group in the traditional sense to measure differences.

## Conclusion

Despite the limitations mentioned above, as one of the few studies on father involvement and children and adolescents mental health problems in rural China, The findings validate the correlation between father involvement and children’s risk of depression and anxiety, establishing that different levels of father involvement predict depression and anxiety in children and adolescents. The present study offers a novel perspective on intervention policies addressing children and adolescents mental health issues. Policymakers, educational authorities, healthcare professionals, and families can encourage father’s participation, enhance the level of fathers’ parenting investment, and thereby protect and promote the mental health of children and adolescents.

## Supporting information

Jiang et al. supplementary material 1 Jiang et al. supplementary material

Jiang et al. supplementary material 2 Jiang et al. supplementary material

## Data Availability

The data used in this study are open and publicly available, free of charge. Data and materials are available at https://doi.org/10.57760/sciencedb.j00001.00464. The analytic Code is available from J.J. The analyses presented here were not preregistered.

## Abbreviations

CDI: Child Depression Inventory
FIQ: Father Involvement Questionnaire
GAD-7: Generalized Anxiety Disorder 7-item
IQR: interquartile range

## Supplementary material

The supplementary material for this article can be found at http://doi.org/10.1017/gmh.2024.70.
