# Father’s involvement associated with rural children’s depression and anxiety: A large-scale analysis based on data from seven provinces in China – CORRIGENDUM

**DOI:** 10.1017/gmh.2025.1

**Published:** 2025-01-27

**Authors:** Jian Jiang, Xuwei Tang, Zhifeng Lin, Yulan Lin, Zhijian Hu

**Keywords:** father involvement, depression, anxiety, children and adolescents, a large-scale analysis

Upon publication of this article the 2nd affiliation for the first author Jian Jiang was omitted

The full and correct affiliations for Jian Jian are:

^1^School of Public Health, Fujian Medical University, Fuzhou, China

^2^Fujian Provincial Hospital, Fuzhou, China

The article has been updated to reflect this.
