# Supplementary material for: Father’s involvement associated with rural children’s depression and anxiety: A large-scale analysis based on data from seven provinces in China
Source: Glob Ment Health (Camb). 2024 Aug 27;11:e71. doi: 10.1017/gmh.2024.70 (PMC11391148; doi:10.1017/gmh.2024.70)
Supplement: Jiang et al. supplementary material 1 — Jiang et al. supplementary material [file S2054425124000700sup001.doc]

Appendix

Father Involvement Questionnaire (FIQ)

The following questions are designed to understand your father's interactions with you. Please select the most appropriate option from 0 to 4 based on the questions.

|  | item | **Never (0)** | **Rarely (1)** | **Sometimes (2)** | **Often (3)** | **Always (4)** |
| --- | --- | --- | --- | --- | --- | --- |
| Interaction | 1. Father urges you to do my homework |  |  |  |  |  |
|  | 2. Father finds time to chat with you |  |  |  |  |  |
|  | 10. Father expresses his feelings to you  through words |  |  |  |  |  |
|  | 13. When you need to see a doctor, father  takes you there |  |  |  |  |  |
|  | 15. Father expresses his emotions to you  through body language |  |  |  |  |  |
|  | 18. Dad takes care of your daily life and  routine |  |  |  |  |  |
|  | 19. Father picks you up and drops you off  at school |  |  |  |  |  |
|  | 20. When you're not feeling well, father  takes care of you |  |  |  |  |  |
| Accessibility | 3. Father corrects your misbehaving  behavior |  |  |  |  |  |
|  | 4. Father goes traveling with you |  |  |  |  |  |
|  | 5. Father exercises with you |  |  |  |  |  |
|  | 6. Father does what you want to do together  with you |  |  |  |  |  |
|  | 7. Father plays with you near our home |  |  |  |  |  |
|  | 8. Father accompanies you to visit  off-campus educational places, such as  science museums, libraries, zoos, and so on |  |  |  |  |  |
|  | 16. Father plays with you at home |  |  |  |  |  |
| Responsibility | 9.Father talks with you about the things that  happen in your life |  |  |  |  |  |
|  | 11.When you encounter difficulties in your  studies, father helps you with the answers |  |  |  |  |  |
|  | 12. Father talks with you about the things  that interest you |  |  |  |  |  |
|  | 14. Father teaches you methods for  studying |  |  |  |  |  |
|  | 17. Father emphasizes the principles of  interacting with others and behaving  appropriately to you, such as being polite to  people |  |  |  |  |  |
|  | 21.Father will discuss with you the  difficulties you encounter in your studies |  |  |  |  |  |
|  | 22. Father educates you to be responsible  for your own affairs |  |  |  |  |  |
