# Supplementary material for: Father’s involvement associated with rural children’s depression and anxiety: A large-scale analysis based on data from seven provinces in China
Source: Glob Ment Health (Camb). 2024 Aug 27;11:e71. doi: 10.1017/gmh.2024.70 (PMC11391148; doi:10.1017/gmh.2024.70)
Supplement: Jiang et al. supplementary material 2 — Jiang et al. supplementary material [file S2054425124000700sup002.doc]

Table 1.

The correlation between scores of each dimension of FIQ and depression and anxiety

|  |  | **Depression Risk** | | | |  | **Anxiety Risk** | | | |
| --- | --- | --- | --- | --- | --- | --- | --- | --- | --- | --- |
|  |  | **OR (95% CI) ^a^** | ***P*** | **OR (95% CI) ^b^** | ***P*** |  | **OR (95% CI) ^a^** | ***P*** | **OR (95% CI) ^b^** | ***P*** |
| Interaction | Low | 1.000 (reference) |  | 1.000 (reference) |  |  | 1.000 (reference) |  | 1.000 (reference) |  |
|  | Medium-low | 0.43(0.24~0.75) | 0.003 | 0.53(0.28~0.98) | 0.043 |  | 0.61(0.23~1.60) | 0.313 | 1.05(0.36~3.03) | 0.931 |
|  | Medium-high | 0.42(0.24~0.73) | 0.002 | 0.68(0.36~1.28) | 0.231 |  | 0.50(0.19~1.37) | 0.180 | 0.96(0.31~2.94) | 0.941 |
|  | High | 0.23(0.13~0.41) | <0.001 | 0.45(0.23~0.87) | 0.018 |  | 0.60(0.24~1.49) | 0.269 | 1.36(0.46~3.99) | 0.574 |
|  |  |  |  |  |  |  |  |  |  |  |
| Accessibility | Low | 1.000 (reference) |  | 1.000 (reference) |  |  | 1.000 (reference) |  | 1.000 (reference) |  |
|  | Medium-low | 0.59(0.34~1.03) | 0.062 | 0.62(0.34~1.15) | 0.129 |  | 0.64(0.25~1/61) | 0.341 | 0.82(0.30~2.19) | 0.685 |
|  | Medium-high | 0.52(0.31~0.87) | 0.012 | 0.66(0.38~1.16) | 0.151 |  | 0.40(0.15~1.04) | 0.059 | 0.51(0.18~1.41) | 0.194 |
|  | High | 0.19(0.10~0.35) | <0.001 | 0.29(0.15~0.57) | <0.001 |  | 0.32(0.12~0.85) | 0.025 | 0.48(0.16~1.45) | 0.194 |
|  |  |  |  |  |  |  |  |  |  |  |
| Responsibility | Low | 1.000 (reference) |  | 1.000 (reference) |  |  | 1.000 (reference) |  | 1.000 (reference) |  |
|  | Medium-low | 0.72(0.42~1.24) | 0.234 | 0.89(0.49~1.62) | 0.712 |  | 1.20(0.49~2.94) | 0.692 | 1.88(0.69~5.14) | 0.218 |
|  | Medium-high | 0.40(0.24~0.69) | <0.001 | 0.55(0.31~0.99) | 0.048 |  | 0.35(0.12~1.04) | 0.060 | 0.58(0.18~1.91) | 0.371 |
|  | High | 0.21(0.11~0.38) | <0.001 | 0.35(0.18~0.69) | 0.002 |  | 0.68(0.27~1.73) | 0.421 | 1.40(0.46~4.27) | 0.557 |

Note: a is a univariate logistic analysis. b is a multivariate logistic analysis and adjusted for factors such as gender, age, parental relationship, whether the child is accompanied by a biological father, whether the child is accompanied by a biological mother, whether the child is accompanied by a stepfather, whether the child is accompanied by a stepmother, whether the child is an only child, the father's education level, the mother's education level, and the level of father involvement.
