# Supplementary material for: Father’s involvement associated with rural children’s depression and anxiety: A large-scale analysis based on data from seven provinces in China
Source: Glob Ment Health (Camb). 2024 Aug 27;11:e71. doi: 10.1017/gmh.2024.70 (PMC11391148; doi:10.1017/gmh.2024.70)
Supplement: Peer review history

# Editor report: Recommendation: Father’s involvement associated with rural children’s depression and anxiety: A large-scale analysis based on data from seven provinces in China — R0/PR2

**DOI:** 10.1017/gmh.2024.70.pr2

**Affiliations:** School of Public Health and Preventive Medicine, Monash University Faculty of Medicine Nursing and Health Sciences, Australia

No accompanying comment.

# Editor report: Decision: Father’s involvement associated with rural children’s depression and anxiety: A large-scale analysis based on data from seven provinces in China — R0/PR3

**DOI:** 10.1017/gmh.2024.70.pr3

**Affiliations:** Johns Hopkins University Bloomberg School of Public Health, United States

No accompanying comment.

# Editor report: Recommendation: Father’s involvement associated with rural children’s depression and anxiety: A large-scale analysis based on data from seven provinces in China — R1/PR5

**DOI:** 10.1017/gmh.2024.70.pr5

**Affiliations:** School of Public Health and Preventive Medicine, Monash University Faculty of Medicine Nursing and Health Sciences, Australia

No accompanying comment.

# Editor report: Decision: Father’s involvement associated with rural children’s depression and anxiety: A large-scale analysis based on data from seven provinces in China — R1/PR6

**DOI:** 10.1017/gmh.2024.70.pr6

**Affiliations:** Johns Hopkins University Bloomberg School of Public Health, United States

No accompanying comment.

# Editor report: Recommendation: Father’s involvement associated with rural children’s depression and anxiety: A large-scale analysis based on data from seven provinces in China — R2/PR8

**DOI:** 10.1017/gmh.2024.70.pr8

**Affiliations:** School of Public Health and Preventive Medicine, Monash University Faculty of Medicine Nursing and Health Sciences, Australia

No accompanying comment.

# Editor report: Decision: Father’s involvement associated with rural children’s depression and anxiety: A large-scale analysis based on data from seven provinces in China — R2/PR9

**DOI:** 10.1017/gmh.2024.70.pr9

**Affiliations:** Johns Hopkins University Bloomberg School of Public Health, United States

No accompanying comment.
